# Effect of *Bifidobacterium breve* in Combination With Different Antibiotics on *Clostridium difficile*

**DOI:** 10.3389/fmicb.2018.02953

**Published:** 2018-12-04

**Authors:** Jingpeng Yang, Hong Yang

**Affiliations:** State Key Laboratory of Microbial Metabolism, School of Life Sciences and Biotechnology, Shanghai Jiao Tong University, Shanghai, China

**Keywords:** *Bifidobacterium breve*, *Clostridium difficile*, antibiotics, toxin production, gene expression

## Abstract

While combinations of probiotics with antibiotics have exhibited beneficial and adverse effects in the treatment of *Clostridium difficile* infection (CDI), no substantive explanation has been provided for these effects. In this study, *C. difficile* ATCC 9689 (CD) was treated with *Bifidobacterium breve* (YH68) in combination with five different antibiotics to explore the effects of the different combinations on *C. difficile*. Cell-free culture supernatant (CFCS) of YH68 was combined with metronidazole (MTR), vancomycin (VAN), clindamycin (CLI), ceftazidime (CAZ) or ampicillin (AMP) to treat CD. The plate counting method was used to determine the growth and spore production of CD, and cell damage was assessed by the measurement of extracellular ATP levels with a luminescence-based kit. The production of toxin A/B was measured with an ELISA kit. The gene expression levels of *tcdA* and *tcdB* in CD were evaluated by real-time qPCR. The CFCS of YH68 (3 × 10^9^ CFU/mL) at 0.25 times the minimal inhibitory concentration (MIC) (0.25YH68) in combination with the five antibiotics exerted stronger inhibitory effects on the growth and spore production of CD than the same antibiotics in the absence of 0.25YH68, except 0.25YH68&MTR&AMP, 0.25YH68&MTR&CAZ, and 0.25YH68&VAN&CLI. However, treatment with 0.25YH68&VAN, 0.25YH68&AMP, 0.25YH68&MTR&CAZ, 0.25YH68&VAN&CAZ, 0.25YH68&VAN&AMP, and 0.25YH68&CAZ&AMP resulted in increased cell damage. In addition, the different combinations, except 0.25YH68&CLI, 0.25YH68&MTR&AMP and 0.25YH68&VAN&CLI, dramatically reduced the production of toxin A/B in comparison with the effects of the same antibiotics in the absence of 0.25YH68. The gene expression levels of *tcdA* and *tcdB* in CD were lowered upon treatment with 0.25YH68 in combination with MTR, CLI, CAZ, MTR&CAZ, MTR&AMP, CLI&CAZ, and CLI&AMP, whereas the levels were enhanced by 0.25YH68 in combination with VAN, AMP, MTR&CLI, VAN&CLI, VAN&AMP, and CAZ&AMP. In summary, YH68 in combination with specific antibiotics could enhance the inhibitory effects of antibiotics against CD. In addition, the antagonistic effects between some antibiotics could be weakened by YH68.

## Introduction

*Clostridium difficile* is a Gram-positive obligate anaerobic bacterium that can produce spores, which are frequently accompanied by a pig-like smell (Smits et al., 2016). As a primary cause of antibiotic-associated diarrhea (AAD), *C. difficile* multiplies rapidly and flourishes in the colon after the diversity of the gut microbiota has been altered by antibiotic therapy (Kociolek and Gerding, 2016). Subsequently, excess toxins A and B are secreted and cause damage to human intestinal tissue (Alcalá Hernández et al., 2017). Diarrhea triggered by *C. difficile* is commonly recognized as *C. difficile* infection (CDI), which accounts for 20–30% of AAD (Xu et al., 2017). The manifestations of CDI vary from mild diarrhea to severe complications associated with pseudomembranous colitis, toxic megacolon and death (Hedge et al., 2008).

Currently, antibiotics, including first-line drugs such as metronidazole (MTR) and vancomycin (VAN), remain the preferred methods of treatment for CDI (Gerber et al., 2008; Kociolek and Gerding, 2016). However, antibiotic therapy can change intestinal microbiota diversity, reduces beneficial bacterial proportion, subsequently generate an environment conducive to *C. difficile* growth, especially for the spore germination (Smits et al., 2016). These spores can survive under the harsh environment for a long time and lead to approximately 25% recurrence of CDI, which is exceedingly difficult to treat in clinical therapy (Korpela et al., 2016). Therefore, alternative treatments to address this challenge are urgently required.

As a new strategy anti-CDI strategy, probiotics have received much attention due to the high degree of safety associated with these strains (Koretz, 2018; Mills et al., 2018). A clinical report demonstrated that administration of probiotics at approximately the same time as the first dose of antibiotic decreased the CDI morbidity (>50%) in hospitalized adults (Shen et al., 2017). Furthermore, previous studies indicated that probiotics combined with specific antibiotics exhibited improved effects on the treatment of CDI (Shen et al., 2017); however, some combinations were not effective, even causing adverse reactions (Golic et al., 2017; Zarandi et al., 2017). Many physicians are thus skeptical of these combinations, mainly due to the lack of a theoretical basis and understanding of the mechanism of action of this type of combination therapy.

In this study, *C. difficile* ATCC 9689 (CD) was treated with *Bifidobacterium breve* (YH68) combined with MTR, VAN, clindamycin (CLI), ceftazidime (CAZ) and ampicillin (AMP). For the first time, we explored the different effects of YH68 combined with these five antibiotics on *C. difficile* to determine the apparent mechanism of action.

## Materials and Methods

### Strains and Growth Conditions

The CD was purchased from the American Type Culture Collection and YH68 (CGMCC No. 14096) was isolated from the feces of healthy individuals (Wang et al., 2017) and provided by Jiaxing Innocul - probiotics Co., Ltd. (Jiaxing, Zhejiang, China). The strains were individually cultured in brain heart infusion (BHI) medium and de Man Rogosa Sharpe broth supplemented with 0.05% (w/v) L-cysteine (MRSC) for 24–48 h at 37°C aerobically (AnaeroGenTM, Oxoid Ltd., Basingstoke, United Kingdom). The presence of the *tcdA* and *tcdB* genes in CD was verified by specific primers as previously described (Persson et al., 2008). The ability of CD to produce toxin A/B was identified by the *C. difficile* Toxin A/B II ELISA Kit as described in the following paragraphs.

### MIC and FIC Values of Antibiotics

Analytical grade MTR, VAN, CLI, CAZ, and AMP were purchased from Macklin (Shanghai, China). The minimum inhibitory concentrations (MICs) and fractional inhibitory concentrations (FICs) of the different antibiotics against CD were determined in triplicate by the microdilution (Garneau et al., 2014) and checkerboard microdilution methods (Song et al., 2003; Mawabo et al., 2015), respectively.

An overnight CD culture (200 μL) was inoculated into 10 mL of fresh prereduced BHI broth and grown at 37°C anaerobically to approximately 1 × 10^6^ CFU/mL and then added to triplicate wells of a 96-well plate containing serial dilutions (0.5–1024 μg/mL) of the different antibiotics. The plates were incubated anaerobically at 37°C for 48 h and then analyzed by using a microplate reader (OD_630_). The MIC was defined as the lowest antibiotic concentrations that inhibited measurable bacterial growth.

The FIC values of different antibiotics used in combination were calculated after the MICs of the different antibiotics were determined.

$$\begin{array}{c} FIC_{i}=FIC(A)+FIC(B)=(A)/(MIC_{A})+(B)/(MIC_{B}) \end{array}$$

Conventionally, FIC values of 0.5 or less, 0.75, 1.0, and 2.0 or more have been defined as partially synergistic, additive, indifferent and antagonistic, respectively (Song et al., 2003; Mawabo et al., 2015; Fratini et al., 2017).

### MIC of *B. breve*

The MIC of the cell-free culture supernatant (CFCS) of YH68 against CD was determined by the method reported by Piotrowski et al. (2017) with some modifications. The CFCS of YH68 was collected from 100 mL of bacterial culture (3 × 10^9^ colony-forming units (CFU)/mL) after centrifugation (12,000 r/min, 10 min) and filtration (0.22 μm). Then, 1:2, 1:4, 1:8, 1:16, 1:32, and 1:64 dilutions of the CFCS of YH68 were prepared. Tubes containing 8 mL of the different dilutions were inoculated with 200 μL of CD bacterial culture (1 × 10^6^ CFU/mL) and incubated at 37°C for 48 h under anaerobic conditions. After 48 h, the turbidity of the cultures was measured as an indicator of bacterial growth. The lowest dilution ratio of the CFCS that showed no turbidity was designated as the MIC.

### Preparation of Different Combinations

When using each antibiotic alone, the concentration of the antibiotic was set to 1 × MIC, e.g., 2 μg/mL MTR. When using combinations of antibiotics, the concentrations of both antibiotics were set to their corresponding FICs, e.g., MTR&VAN (0.25 and 0.5 μg/mL, respectively). When using YH68 combined with antibiotics, the YH68 and antibiotic concentrations were set to 0.25 × MIC and 1 × MIC/FIC, respectively, e.g., 0.25YH68&MTR (0.25 × MIC of YH68 and 2 μg/mL of MTR). Fresh BHI broth lacking CD bacterial culture was regarded as a negative control, and BHI broth with CD but lacking antibiotics or YH68 was regarded as a positive control.

### Growth and Spore Production

CD was cultured anaerobically to log phase in BHI broth and then divided into different groups (Aldape et al., 2015). Then, 1 mL samples were removed from each group at 0, 6, 12, 24, and 48 h. One half (500 μL) of each sample was used to determine viable CFU/mL. The remaining 0.5 mL of each sample was collected to measure spore production. Samples were mixed with 0.5 mL of 100% ethanol for 1 h with rotation at 4°C to kill the vegetative cells (Lawley et al., 2009). Then, the cell pellets were collected by centrifugation (12,000 r/min for 5 min) and washed twice in PBS (pH 7.4). Following the final wash, the pellets were resuspended in 0.5 mL of PBS. Spores were enumerated by serially diluting the samples in PBS and plating onto BHI agar plates. All these plates were incubated at 37°C anaerobically for 72 h, and the resultant CFU/mL values were deemed to represent the relative number of viable spores produced.

### Cell Damage

The integrity of the cell membrane can be damaged by antibiotics or YH68, accompanied by leakage of intracellular ATP. Therefore, changes in extracellular ATP levels can accurately reflect cell damage. The extracellular ATP level was measured at appropriate time intervals by a previously described bioluminescence-based method (Suzuki et al., 2005) using a microplate reader (PE EnSpire 2300) and a luminescence kit (CellTiter-Glo^®^ 2.0 Kit, Promega, United States).

### Toxin Production and Gene Expression

Two hundred microliters of fresh CD bacterial culture was added to the different groups and cultured anaerobically at 37°C for 3 h. Subsequently, 1 mL samples were removed from each group at 0, 6, 12, 24, and 48 h. The cell pellets were collected by centrifugation (12,000 r/min) and used to prepare total RNA for PCR analysis of gene expression. The CFCSs were filter sterilized (0.22 μm) to assess soluble TcdA/B production using an ELISA kit.

The levels of toxin proteins A and B in the CFCSs of CD were measured in combination (i.e., toxin AB) using the *C. difficile* Toxin A/B II ELISA Kit (Runyu Ltd., Shanghai, China) according to the manufacturer’s instructions. Samples were diluted when necessary to obtain readings within the linear range of the standard (3–3000 ng/mL). All samples were tested in triplicate.

RNA was isolated from the collected CD cell pellets using the RNA-prep Pure Kit for Bacteria (TianGen Biotech, Beijing, China) according to the manufacturer’s instructions. Contaminant genomic DNA was removed by two rounds of DNase treatment (DNA-free Kit; Tiangen), and the final RNA yield and quality were assessed by ultraviolet absorbance measurement and agarose gel electrophoresis, respectively. Changes in *tcdA* and *tcdB* gene expression levels were assessed by real-time qPCR using a method reported by Aldape et al. (2015) with some modifications; the method has been described below with specific primers.

cDNA was synthesized from each sample (500 ng) using All-in-One First-Strand cDNA Synthesis SuperMix (TransGen Biotech, Beijing, China) and amplified using TransStart Top Green qPCR SuperMix (TransGen Biotech) in a Mastercycler ep realplex system (Eppendorf, Hamburg, Germany) as follows: 30 s at 94°C, followed by 45 cycles at 94°C for 5 s, 55°C for 15 s and 72°C for 10 s. *C. difficile* 16S rRNA served as the internal control. Relative gene expression was determined using the 2^-ΔΔCt^ method. The sample mean Ct of the 16S rRNA (internal control gene) was subtracted from the sample mean Ct of the *tcdA* and *tcdB* genes (ΔCt). The ΔCt of the untreated control at 6 h was subtracted from the mean ΔCt of each experimental sample (ΔΔCt). This 2^-ΔΔCt^ method provides the fold change in gene expression of the gene of interest normalized to the expression of the 16S rRNA internal control and relative to the untreated control at 6 h.

### Statistical Analysis

Each assay was performed in triplicate, and the data are expressed as the means ± standard deviations. The differences between the groups were examined by one-way ANOVA using Minitab 16.2.3 software (Minitab Inc., State College, PA, United States). A *P*-value of <0.05 was considered statistically significant.

## Results

### MICs and FICs

The MICs of MTR, VAN, CLI, CAZ, and AMP were determined for CD by the microdilution method. CD showed different reaction to the five antibiotics, with MIC values of 2, 1, 32, 8, and 32 μg/mL to MTR, VAN, CLI, CAZ, and AMP, respectively (Table 1). The FICs of the antibiotics used in combination were determined by the checkerboard microdilution method, and the results are shown in Table 2. Partially synergistic (0.5 < FIC ≤ 0.75), additive (0.75 < FIC ≤ 1.0), indifferent effects (1.0 < FIC ≤ 2.0), and antagonistic effects (FIC > 2.0) were observed in this study.

**Table 1:** Minimal inhibitory concentration (MICs) of the five antibiotics.

Antibiotics^1^	MIC (μg/mL)
MTR	2
VAN	1
CLI	32
CAZ	8
AMP	32

*^*1*^MTR, metronidazole; VAN, vancomycin; CLI, clindamycin; CAZ, ceftazidime; AMP, ampicillin*.

**Table 2:** Fractional inhibitory concentrations (FICs) of the five antibiotics.

Antibiotics^1^	FICs (μg/mL)	Effect
MTR&VAN	0.625 (0.25, 0.5)	Partially synergistic
MTR&CLI	3 (2, 64)	Antagonistic
MTR&CAZ	2 (2, 8)	Indifferent
MTR&AMP	2 (2, 32)	Indifferent
VAN&CLI	2.125 (0.125, 64)	Antagonistic
VAN&CAZ	1.0625 (0.0625, 8)	Indifferent
VAN&AMP	1.25 (0.25, 32)	Indifferent
CLI&CAZ	4 (64, 16)	Antagonistic
CLI&AMP	1.125 (4, 32)	Indifferent
CAZ&AMP	1 (4, 16)	Additive

*^*1*^MTR, metronidazole; VAN, vancomycin; CLI, clindamycin; CAZ, ceftazidime; AMP, ampicillin*.

The MIC of the CFCS of YH68 was measured by microdilution, and the double-dilution ratio of the CFCS (3 × 10^9^ CFU/mL) was recognized as the MIC.

### Effects of Different Combinations on the Growth and Spore Production of CD

**FIGURE 1:**
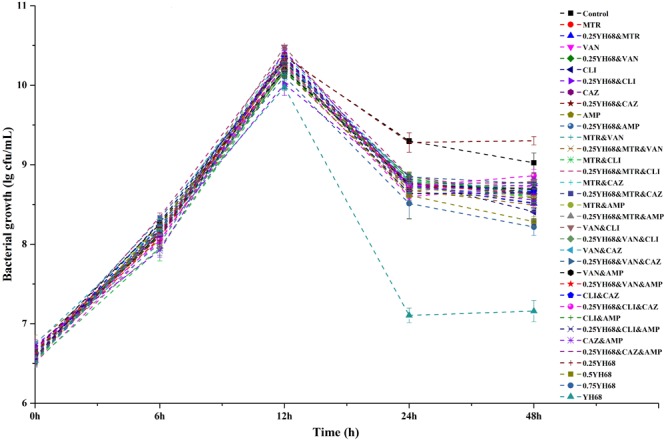
Effects of different combinations on the growth of *Clostridium difficile* ATCC 9689.

**FIGURE 2:**
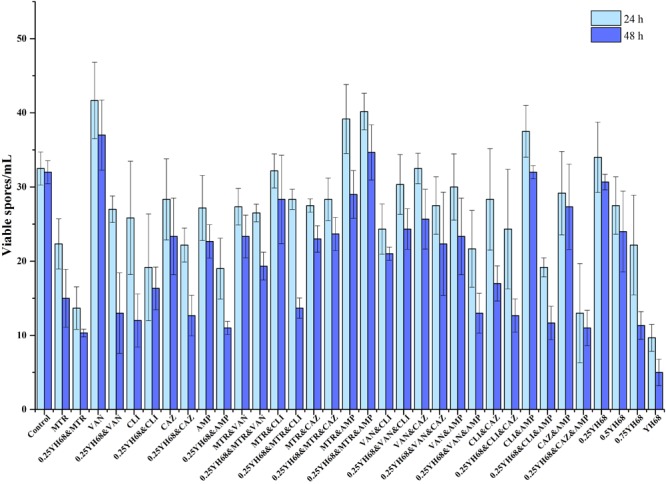
Effects of different combinations on the spore production of *C. difficile* ATCC 9689.

The growth curves of CD in all the groups exhibited an exponentially increasing trend, and the cell densities of these groups were not significantly different (*p* < 0.05) for the first 12 h of growth (Figure 1). After 12 h, the CFU per mL in all the groups decreased rapidly, especially that of the YH68 group, which had the lowest values at 24 h (7.11 ± 0.09 lgCFU/mL) and 48 h (7.16 ± 0.14 lgCFU/mL). Overall, both the antibiotic-treated groups and groups treated with 0.25YH68 in combination with antibiotics exerted stronger inhibitory effects on the growth of CD than the positive control (9.30 ± 0.03 lgCFU/mL at 24 h, 9.02 ± 0.12 lgCFU/mL at 48 h); the group treated with 0.25YH68 alone exhibited a slight increase. Formation of viable spores was observed, and the data in Figure 2 indicate that few spores (less than 50 spores/mL) were produced by CD in all the groups after 24 h, and the number of spores at 48 h was less than that at 24 h. In particular, among all these groups, the YH68 group exhibited the greatest inhibition of spore production (less than 10 spores/mL). Notably, some antibiotics used alone or in combination, such as VAN and CLI&AMP, actually stimulated the production of spores. In addition, 0.25YH68 alone had no inhibitory effect on the growth and spore production of CD, but when 0.25YH68 was combined with some antibiotics, a stronger inhibitory effect on CD spore production was observed than that observed with the same antibiotics in the absence of 0.25YH68.

### Effects of Different Combinations on the Extracellular ATP Level of CD

**FIGURE 3:**
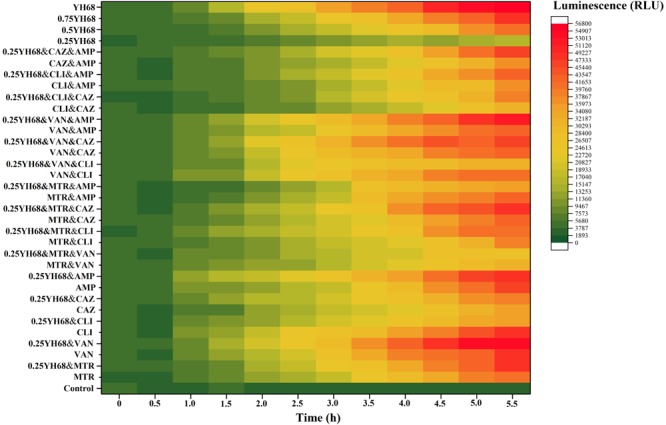
Effects of different combinations on extracellular ATP levels of *C. difficile* ATCC 9689.

The change in the extracellular ATP level was visualized as a heat map. The luminescence value was regarded as an index and depicted in shades of green or red. The redder the color was, the higher the ATP level. As shown in Figure 3, the levels of extracellular ATP in all the groups exhibited a steady increasing trend over 5.5 h, except for that in the control (without antibiotics or YH68). A relatively high level of extracellular ATP was found in the VAN, CLI, AMP, 0.75YH68, YH68, 0.25YH68&MTR, 0.25YH68&VAN, 0.25YH68&AMP, 0.25YH68&MTR&CAZ, 0.25YH68&VAN&CAZ, 0.25YH68&VAN&AMP and 0.25YH68&CAZ&AMP groups at 5.5 h, indicating that these groups caused more severe cell damage to CD than the other groups and induced further outflow of intracellular ATP. These results suggest that these combinations exhibit the strongest antibacterial activity among all these groups. The extracellular ATP level of CD did not increase dramatically when the cells were treated with 0.25YH68 alone. However, when 0.25YH68 was used in combination with some antibiotics, e.g., 0.25YH68&VAN vs. VAN, 0.25YH68&VAN&AMP vs. VAN&AMP, the overall damage caused to CD cells was prominent.

### Effects of Different Combinations on Toxin Production by CD

**FIGURE 4:**
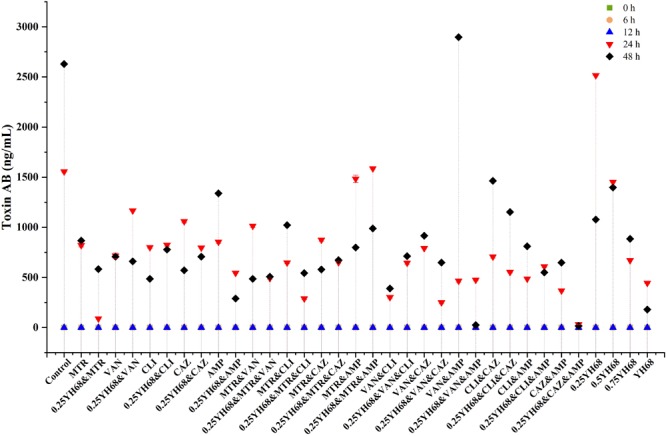
Effects of different combinations on toxin A/B production by *C. difficile* ATCC 9689.

The toxin proteins A and B produced by CD were detected by ELISA after 24 h of growth (Figure 4). The production of toxin A/B in the presence of all these antibiotics or 0.25YH68 combined with the antibiotics (except VAN&AMP and 0.25YH68) was lower than that observed for the positive control (1556.38 ± 7.24 ng/mL at 24 h, 2628.74 ± 3.62 ng/mL at 48 h). Specifically, the levels of toxin A/B in cells treated with MTR, VAN, CLI, CAZ, AMP, 0.25YH68&MTR, 0.25YH68&VAN, 0.25YH68&CLI, 0.25YH68&CAZ, 0.25YH68&AMP, MTR&VAN, and 0.25YH68&MTR&VAN were lower by 50% than that in the positive control. Nevertheless, notably, the levels of toxin A/B in some antibiotic-treated groups (AMP, MTR&CLI, and VAN&AMP) decreased at 24 h but increased at 48 h. Additionally, the combinations of two antibiotics achieved synergistic effects that exceeded the inhibitory effects of single antibiotics on toxin production, as observed, for example, for the toxin production levels in the groups treated with MTR (867.92 ± 3.62 ng/mL), VAN (706.68 ± 0.00 ng/mL) and MTR&VAN (486.57 ± 7.24 ng/mL) at 48 h. However, a few combinations exhibited the opposite trend, as observed, for example, for the toxin production levels in the groups treated with CLI (486.57 ± 7.24 ng/mL), CAZ (571.03 ± 3.62 ng/mL) and CLI&CAZ (1464.24 ± 7.24 ng/mL). Interestingly, 0.25YH68 in combination with most antibiotics achieved a better inhibitory effect on the production of toxin A/B than that achieved by the same antibiotics without 0.25YH68, e.g., toxin production in the AMP group was 1338.84 ± 10.86 ng/mL, but the value decreased rapidly to 289.50 ± 3.62 ng/mL when 0.25YH68 was added. A similar significant difference was also observed between MTR&CLI (1021.48 ± 10.86 ng/mL) and 0.25YH68&MTR&CLI (542.88 ± 7.24 ng/mL) at 48 h.

### Effects of Different Combinations on Gene Expression in CD

**FIGURE 5:**
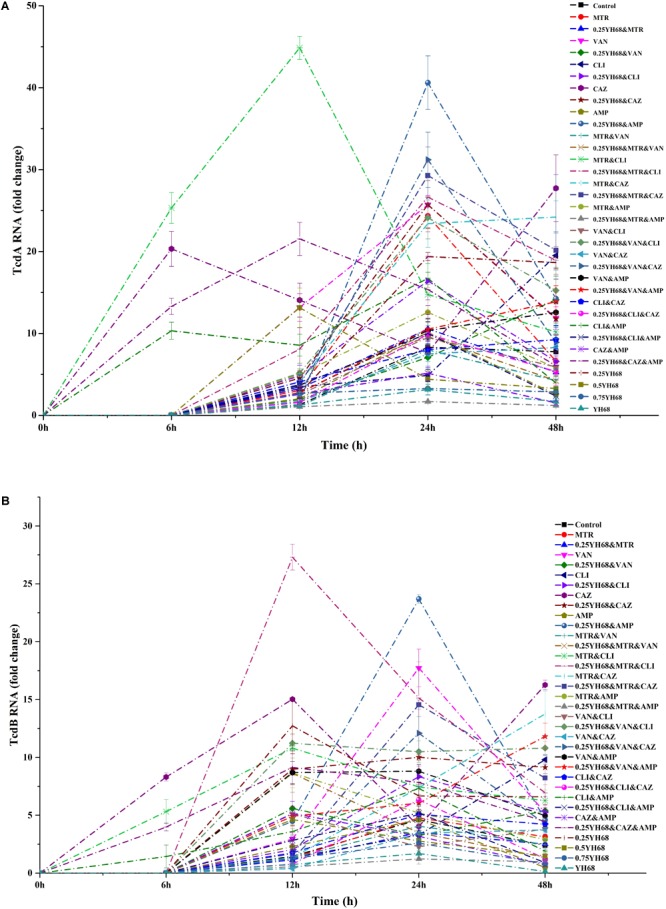
Effects of different combinations on toxin gene expression in *C. difficile* ATCC 9689 (**A**: tcdA, **B**: tcdB).

The gene expression of *tcdA* and *tcdB* began before the production of toxins A and B (Figures 4, 5). The expression levels of both genes were different among all these groups.

Some of the antibiotics or 0.25YH68 combined with antibiotics induced the upregulation of *tcdA* (Figure 5A), e.g., the expression level of *tcdA* in the CAZ group was enhanced approximately 20-fold in comparison with that of the positive control at 48 h. some similar results were also obtained for the VAN, CLI and 0.25YH68&VAN groups. In contrast, some groups, such as 0.25YH68&MTR&AMP, exhibited decreased *tcdA* expression. The fold change in *tcdA* expression in the 0.25YH68&MTR&AMP group decreased by approximately sevenfold in comparison with that of the positive control at 48 h. Similar results were also obtained for the MTR, AMP, and 0.25YH68&MTR groups. Interestingly, 0.25YH68 combined with some antibiotics achieved stronger inhibition of the gene expression of *tcdA* than that achieved by the same antibiotics without 0.25YH68, as observed, for example, for the CLI (19.5-fold at 48 h) and 0.25YH68&CLI (6.6-fold at 48 h) groups. However, a few antibiotics exhibited enhanced gene expression of *tcdA* when combined with 0.25YH68, as observed, for example, for the VAN (11.9-fold) and 0.25YH68&VAN (14.1-fold) groups. In addition, upon increasing the concentration of YH68 to 1 × MIC, the inhibitory effects on the expression of *tcdA* increased, as observed, for example, for 0.25YH68 (18.7-fold), 0.5YH68 (3.3-fold), 0.75YH68 (2.9-fold), and YH68 (1.7-fold) at 48 h.

The gene expression of *tcdB* in some groups was also enhanced (Figure 5B). At 48 h, the gene expression of *tcdB* in the CAZ group was enhanced approximately 14-fold in comparison with that of the positive control, and similar changes were also observed for the VAN, CLI and 0.25YH68&VAN groups. In contrast, some groups exhibited decreased *tcdB* expression, such as 0.25YH68&MTR&AMP. The gene expression of *tcdB* in the 0.25YH68&MTR&AMP group was reduced by approximately twofold in comparison with that of the control at 48 h. A similar trend was also observed for the MTR, AMP, and 0.25YH68&MTR groups. Interestingly, 0.25YH68 combined with some antibiotics achieved stronger inhibition of the gene expression of *tcdB* than that achieved by the same antibiotics without 0.25YH68, for example, for the CLI (9.8-fold) and 0.25YH68&CLI (5.3-fold) groups at 48 h. However, a few antibiotics enhanced the gene expression of *tcdB* when combined with 0.25YH68, as observed, for example, for the AMP (0.5-fold) and 0.25YH68&AMP (4.6-fold) groups at 48 h. With increasing concentrations of YH68 alone, the inhibition of the expression of *tcdB* increased, as observed, for example, for 0.25YH68 (6.6-fold), 0.5YH68 (1.5-fold), 0.75YH68 (0.8-fold), and YH68 (0.1-fold).

Overall, there was no correlation between the gene expression of *tcdA* and *tcdB* over time; meanwhile, the expression level of *tcdA* was higher than that of *tcdB* at the same time points. Moreover, 0.25YH68 combined with specific antibiotics could enhance the inhibition of gene expression.

## Discussion

The CDI is closely associated with the outbreaks of many severe diseases, and even with high mortality rates (Xu et al., 2017). A report from the United States Centers for Disease Control and Prevention (CDC) revealed that 453,000 patients had CDI, with 29,300 attributable deaths, in the United States in 2011 (Lessa et al., 2015). More seriously, a growing body of evidence suggests this trend is constantly spreading and bringing heavy economic burdens in many areas (Aptekorz et al., 2017; Lebwohl et al., 2017; Xu et al., 2017; Carman et al., 2018; Dong et al., 2018; Liu et al., 2018; Seugendo et al., 2018). The virulence of most *C. difficile* strains depends upon the gene expression of the *tcdA*-encoded toxin A, which is an enterotoxin, and the *tcdB*-encoded toxin B, which is a cytotoxin (Davis et al., 2016). These two toxins can cause intestinal inflammation and neutrophil infiltration in the infected foci, which can ultimately lead to intestinal damage (Roshan et al., 2018).

A report from Liu et al. (2018) showed that the molecular characterization of *C. difficile* isolates in China from 2010 to 2015 is diversity, and ribotype 001 (9689) is one of the most prevalent. Therefore, we chosen *C. difficile* ATCC 9689 as a reference strain in our study. The present study found that *C. difficile* ATCC 9689 (CD) showed different reaction to all the five antibiotics tested. MTR and VAN had the lowest MICs (2 and 1 μg/mL, respectively) among these antibiotics (Table 1), directly indicating the importance of these antibiotics in CDI treatment (Gerber et al., 2008; Lam et al., 2018). Different antibiotics are frequently used in combination during clinical therapy in hopes of achieving the best therapeutic effects (Beinortas et al., 2018; Guh and Kutty, 2018; Ziolkowski et al., 2018). However, some antibiotics used in combination can have side effects and exacerbate the CDI (Zarandi et al., 2017). Data from our study showed that some antibiotics used alone or in combination actually stimulated the production of CD spores, and this phenomenon is similar to the result reported by Aldape et al. (2015). Some evidences indicated that the formation of CD spores is associated with *spo0A* gene transcription in CD, and some antibiotics at certain concentration can increase *spo0A* transcription, which may induce much more spore production. The impact of antibiotics on *C. difficile* sporulation can also vary depending on the different strains of *C. difficile* and the different antibiotics (and its specific mode of action) (Garneau et al., 2014; Aldape et al., 2015). Evaluation of FIC values of these five antibiotics in our study suggested that partially synergistic, additive, antagonistic and indifferent effects exist among all of the antibiotics. Furthermore, these four relationships were directly reflected in the toxin production observed when CD was treated with different combinations. The production of toxins A and B was significantly suppressed by most antibiotics when use alone or in combination in comparison with the effect of the control (2628.74 ± 3.62 ng/mL). However, a few combinations stimulated the toxin production, such as VAN&AMP (2897.47 ± 7.24 ng/mL). Zarandi et al. (2017) found that VAN combined with CLI significantly inhibited the toxin production by *C. difficile*, but the combination of CAZ with VAN or CLI greatly induced the production of toxin A/B. Gerber et al. found that antibiotic-induced toxin production in *C. difficile* isolates is associated with a shift in toxin production to earlier growth stages, along with an upregulation of *tcdA* and *tcdB* gene expression (Gerber et al., 2008). Several studies concluded that antibiotic-induced increases in the toxin production of *C. difficile* were part of a stress-induced response (Honda et al., 1983; Drummond et al., 2003; Gerber et al., 2008; Aldape et al., 2013). Different fluctuations in the timing, magnitude and onset of antibiotic-induced stress responses vary between *C. difficile* isolates and antibacterial stress and these effects may only reflect strain-dependent responses to these antibiotics (Aldape et al., 2015). This finding demonstrated that not all these antibiotics used in combination can help treat CDI. In addition, this finding explains, in terms of toxin production, why some antibiotics used in combination exacerbate diarrhea, as observed in outbreaks of recurrent CDI (rCDI).

The CFCS of *B. breve* (YH68-CFCS) was used in combination with different antibiotics to treat CD in the current study. The concentration of YH68-CFCS was set as 1/4 × MIC (0.25YH68), which was used in combination with different antibiotics to investigate whether 0.25YH68 can promote the inhibitory effects of antibiotics on CD compared to the effects observed when the antibiotics are used alone. Statistical analysis of growth and spore production indicated that the cell densities (24 and 48 h) of CD cultures treated with 0.25YH68 in combination with most of the antibiotics were lower than those of cultures treated with the same antibiotics in the absence of 0.25YH68. For example, a significant difference in spore production was observed between the VAN (37 spores/mL) and 0.25YH68&VAN (13 spores/mL) groups at 48 h. These results suggest that there is a synergistic effect between 0.25YH68 and specific antibiotics on the inhibition of CD. A similar phenomenon was also reflected in the leakage of intracellular ATP from CD upon treatment with different combinations (Figure 3). On the other hand, 0.25YH68 used alone had no inhibitory effect on CD, including on cell density, spore production and toxin production, but the inhibiting effect was enhanced by increasing CFCS concentrations (0.25YH68 < 0.5YH68 < 0.75YH68 < YH68), indicating that 0.25YH68 contained fewer antibiotic substances than the other concentrations, most likely due to dilution. Several studies indicated that there is an antagonism relationship between *bifidobacterium* sp. and some pathogens, and organic acids secreted by *bifidobacterium* may play a crucial role in the antibacterial activity (Delcaru et al., 2016; Valdes-Varela et al., 2016; Wei et al., 2018). Wei et al. (2018) found that CFCS of *B. longum* JDM301 resulted in the degradation of *C. difficile* toxin, especially the degradation of TcdA through the regulation of acid pH (original pH = 4.9). This result suggested that the acid pH induced by probiotics could inhibit the growth of *C. difficile*, as well as it could directly promote the degradation of clostridial toxin (Wei et al., 2018). Our study showed that the antibacterial activity of original CFCS produced by YH68 against CD is strong, which is most likely to the organic acids effect. In addition, CD spores were detected after 24 h in all the groups at a low yield (less than 100 spores/mL). This observation was also consistent with data from previous studies and was mainly associated with the metabolic level of the 9689 strain itself (Garneau et al., 2014; Aldape et al., 2015).

Two or more antibiotics are commonly used in combination in clinical therapy to address not only CDI, but also other conditions, such as postoperative infections (Cohen et al., 2017; Guh and Kutty, 2018; Kunutsor et al., 2018; Takoudju et al., 2018). Therefore, some types of antibiotics have to be used in combination even though the combination may exacerbate CDI (or lead to rCDI). Interestingly, the data in our study demonstrated that the antagonistic effect between some antibiotics was weakened by 0.25YH68. Compared with the effects of antibiotics in the absence of 0.25YH68, toxin production in the presence of 0.25YH68 combined with the same antibiotics was reduced significantly, e.g., toxin production in the MTR&CLI group (the FIC effect of which was antagonistic) was 1021.48 ± 10.86 ng/mL, which was higher than that of the MTR (867.92 ± 3.62 ng/mL) or CLI (486.57 ± 7.24 ng/mL) groups, indicating that toxin production was stimulated by MTR&CLI. However, when 0.25YH68 was combined with MTR&CLI (0.25YH68&MTR&CLI), toxin production decreased to 542.88 ± 7.24 ng/mL, which was almost half of that observed for the MTR&CLI group. This result revealed that YH68 in combination with specific antibiotics might inhibit the adverse effects of CDI without affecting the efficacy of the drug in clinical therapy. In other words, YH68 combined with antibiotics might decrease the recurrence rate of CDI, reduce the side effects of antibiotics, and shorten the recovery period for patients.

The virulence genes *tcdA* and *tcdB*, as two core elements of CD, control the production of toxins. In this study, the expression of *tcdA* and *tcdB* began before the production of toxin A/B. There was no significant correlation between gene expression levels and toxin production over time. Previous reports have demonstrated that gene expression occurs prior to protein production (Stevens et al., 2007; Aldape et al., 2013; Aldape et al., 2015) and two potential mechanisms could explain this phenomenon. First, a certain concentration of antibiotics induced early *tcdA* and *tcdB* transcript production in *C. difficile* and increasing their half-lives until their translation in the stationary phase (Aldape et al., 2013).

Second, toxin proteins (TcdA and TcdB) remain intracellular after translation during the logarithmic phase, subsequently are released once organisms have entered the stationary phase. This action mode seems like TcdE, a protein that is expressed in the stationary phase and is thought to facilitate TcdA and TcdB release through permeabilization of the *C. difficile* cell wall (Tan et al., 2001).

In this study, we found that antibiotics used alone or in combination also upregulated or downregulated the gene expression levels of *tcdA* and *tcdB*. Previous studies suggested that some pathogens sense and respond to a certain concentration of antibiotics with a putative SOS response that includes the up-regulation or down-regulation of virulence-associated genes (Drummond et al., 2003; Stevens et al., 2007; Gerber et al., 2008; Aldape et al., 2013). The fluctuation in gene expression levels vary between different *C. difficile* isolates and different antibiotic challenges (Aldape et al., 2015). Interestingly, for some antibiotics that could inhibit the expression of virulence genes, 0.25YH68 could enhance the inhibitory effect, as observed, for example, for CLI (*tcdA* expression was reduced 19.5-fold, *tcdB* expression was reduced 9.8-fold) and 0.25YH68&CLI (*tcdA* expression was reduced 6.6-fold, *tcdB* expression was reduced 5.3-fold) at 48 h. It seems like some antagonistic antibiotics induced the enhancement of gene expression, whereas this effect was weakened by 0.25YH68, as observed, for example, for CLI&CAZ (*tcdA* expression was induced 9.2-fold, *tcdB* expression was induced 4.2-fold) and 0.25YH68&CLI&CAZ (*tcdA* expression induced was 5.3-fold, *tcdB* expression was induced 1.3-fold).

Given the importance of gut microbial diversity, the use of probiotics to treat CDI is widely recognized by the public because most probiotics are designated as generally recognized as safe (GRAS) bacteria (Leite et al., 2015). Some specific probiotics can not only treat disease, but also regulate the intestinal flora, further increasing the bacterial diversity and improving immunity (Quin et al., 2018; Zhao et al., 2018). A report by Shen et al. (2017) showed that the incidence of CDI in a probiotic-treated cohort (1.6%) was lower than in that in the controls (3.9%) (*P* < 0.001). The combined relative risk of CDI in probiotic users was 0.42 (95% confidence interval, 0.30–0.57; *I*^2^ = 0.0%). In addition, probiotics were significantly more effective if administered at approximately the same time as the first antibiotic dose, with a decrease in efficacy observed for every day of delay in the start of probiotic treatment (*P* = 0.04); probiotics given within 2 days of antibiotic initiation were associated with a greater reduction of the risk for CDI (relative risk, 0.32; 95% confidence interval, 0.22–0.48; *I*^2^ = 0%) than that observed upon later administration (relative risk, 0.70; 95% confidence interval, 0.40–1.23; *I*^2^ = 0%) (*P* = 0.02). There was no increased risk for adverse events among patients administered probiotics. These data suggested that probiotics combined with antibiotics were highly effective in the treatment of CDI; however, it was also reported that not all probiotics play a therapeutic role in the treatment of CDI (Mills et al., 2018). Furthermore, the mechanism by which probiotics combined with antibiotics have (or do not have) a therapeutic effect on CDI has not been fully elucidated.

In the present study, we explored the different effects of YH68 in combination with five antibiotics on CD. Changes in growth, spore production, cell damage, toxin production and gene expression levels were determined to provide a reference for precision medication for CDI and proved the potential value of YH68 combined with these five antibiotics in the clinical therapy. Combinations, such as 0.25YH68&MTR, 0.25YH68&VAN, 0.25YH68&MTR&VAN, 0.25YH68&VAN&AMP, and 0.25YH68&CLI&AMP can be considered and recommended in clinical therapy of CDI.

## Author Contributions

JY and HY designed the experiments. JY performed the experiments. JY and HY analyzed the data and wrote the manuscript.

## Conflict of Interest Statement

The authors declare that the research was conducted in the absence of any commercial or financial relationships that could be construed as a potential conflict of interest.
